# Systematic Map of Chemical and Biological Surfactant Effects on Oxygen Mass Transfer at the Air–Water Interface

**DOI:** 10.1002/wer.70271

**Published:** 2026-01-14

**Authors:** Luciano de Oliveira, Diana Rosa dos Reis, Sérgio Botelho de Oliveira, Klebber Teodomiro Martins Formiga

**Affiliations:** ^1^ Post‐Graduation Program in Environmental Sciences Federal University of Goiás Goiânia Goiás Brazil; ^2^ Post‐Graduation Program in Technology, Management and Sustainability Federal Institute of Goiás Goiânia Goiás Brazil

**Keywords:** biosurfactants, environmental chemistry, gas–liquid interface, kL, kLa, oxygen transfer rate, water treatment

## Abstract

This review summarizes scientific studies from 1963 to 2024 on how chemical and biological surfactants affect oxygen transfer at the air–water interface. Surfactants, which often enter water from human activities, can alter water surfaces and involve the transfer of oxygen, an important aspect of water quality and treatment. We reviewed 54 peer‐reviewed studies and sorted them by surfactant type, water type, and experimental scale. A lot of research has been done on chemical surfactants, but less on biosurfactants. Most of the experiments were conducted in labs, indicating that more field research is needed. There are still more than 92.22% of possible combinations of surfactants and water that have not been tested. Surfactants usually make it harder for oxygen to move through water, but the extent to which they do depends on their chemistry, the amount present, and the water's cleanliness. Research is segregated into distinct disciplines, exhibiting minimal collaboration. This review highlights areas where further research is needed, especially on biosurfactants and their behavior in real‐world water. It also offers ideas for improving wastewater treatment. Our findings support green chemistry and give a framework for better managing oxygen transfer and surfactant pollution in water systems.

## Summary

- Chemical surfactants (e.g., petroleum derivatives) reduce oxygen transfer at the water–air interface via insulating barriers, damaging ecosystems.
- Biodegradable and less toxic biosurfactants can improve reoxygenation compared to chemicals, making them a viable alternative in contaminated environments.
- There is a lack of robust comparative assessment between surfactants on oxygen; research in diverse environments is essential for environmental guidelines.

## Graphical abstract

This systematic review confirms that surfactants reduce oxygen transfer (kL/kLa), with concentration and hydrodynamics being critical factors. There was little diversity in the surfactants tested, and aquatic conditions were simplified, and variables (pH, viscosity) were neglected. Future research should test different surfactants, complex waters, and omitted variables for practical applications (WWTPs, industry).

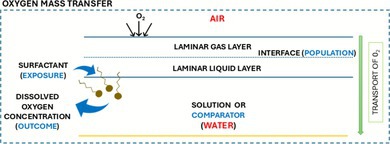

## 1 Introduction

Surfactants are amphiphilic (having both hydrophobic and hydrophilic regions) organic compounds ubiquitous in aquatic environments. They originate from both anthropogenic sources (such as detergents, industrial effluents, and agricultural runoff) and natural biological processes (such as microbial biosurfactants and phytoplankton exudates).

These surface‐active substances accumulate preferentially at air–water interfaces, where they modify interfacial properties, particularly by reducing surface tension, which is the elastic tendency of the interface that makes it acquire the least surface area possible and consequently alter gas transfer dynamics. Given that oxygen dissolution represents a critical process sustaining aquatic ecosystems and engineering systems alike, the understanding of how surfactants influence oxygen mass transfer coefficients k_L_ (the liquid‐phase mass transfer coefficient) and k_L_a (the overall oxygen transfer coefficient, including surface area and time) is a point to consider for environmental management, water quality modeling, and wastewater treatment optimization (Ferreira et al. 2020; Arora and Keshari 2021).

Early foundational work showed that surfactants reduce oxygen transfer rates by damping interfacial turbulence (reducing movement at the air–water boundary) and creating diffusional barriers (hindering the movement of gas molecules) at contaminated interfaces (Eckenfelder 1970; Downing and Truesdale 1955).

Seminal studies demonstrated that organic contaminants substantially decrease aeration efficiency—the rate at which oxygen is transferred from air to water—in activated sludge systems (Hwang and Stenstrom 1985; Rosso and Stenstrom 2006). Subsequent research quantified this effect using the alpha factor (α), defined as the ratio k_L_a in contaminated water to that in clean water (*α* = k_L_a contaminated/k_L_a clean), with the alpha factor typically ranging from 0.3 to 0.9 in wastewater treatment plants, depending on surfactant loading and operational conditions (Rosso, Huo, and Stenstrom 2006; McClure et al. 2017).

Despite this established knowledge base for chemical surfactants (particularly anionic compounds like sodium dodecyl sulfate [SDS]), we have identified a critical gap: biosurfactants, which are structurally distinct microbial metabolites with lower toxicity and environmental persistence, remain vastly understudied regarding their effects on oxygen transfer at air–water interfaces (van der Meer et al. 1992; Bai et al. 2024).

This knowledge gap is particularly problematic given the growing environmental relevance of biosurfactants, surface‐active compounds produced by microorganisms. Microbial surface‐active compounds are naturally produced during hydrocarbon biodegradation, occur in eutrophic waters, and are being explored as “green” alternatives to synthetic surfactants in industrial applications (Banat et al. 2010; Datta et al. 2024).

Yet researchers have only partially explained the mechanisms by which biosurfactants—with their diverse molecular structures (such as glycolipids, lipopeptides, and phospholipids)—influence interfacial oxygen transport. Moreover, existing reviews have primarily examined chemical surfactants in controlled laboratory settings using tap or purified water and have paid limited attention to environmentally relevant matrices (natural freshwater and seawater) or to field‐scale observations (Jamnongwong et al. 2010).

Systematic mapping is a good way to handle this scattered research. It differs from traditional narrative reviews by using clear, repeatable steps to search and screen studies. This lets us thoroughly catalog research, spot clusters of knowledge, and identify unknown topics without combining effect sizes (James et al. 2016; Collaboration for Environmental Evidence 2022). We believe this approach works well in cases like surfactant effects on oxygen transfer, where studies differ in design, surfactant type, and reported outcomes.

This paper aims to conduct a systematic map answering the primary question: What evidence exists on the impact of chemically and biologically synthesized surfactants on oxygen mass transfer at the air–water environmental interface? Specifically, we seek to (1) characterize the distribution of research across surfactant types (chemical vs. biological), experimental populations (water matrices), and outcome variables (k_L_, k_L_a, α‐factors); (2) identify methodological approaches and geographic/institutional research clusters; and (3) reveal critical knowledge gaps—particularly regarding biosurfactants, complex environmental waters, and field‐scale studies—to guide future research priorities.

By systematically mapping this evidence landscape spanning six decades (1963–2024), we provide a foundation for targeted meta‐analyses, improved predictive models for environmental oxygen dynamics, and evidence‐based optimization of aeration technologies in water treatment systems. This synthesis addresses the urgent need to connect laboratory‐scale mechanistic findings with field‐applicable knowledge, especially as “green chemistry” policies increasingly promote biosurfactant alternatives without adequate understanding of their impacts on critical ecosystem processes, such as oxygen reaeration.

## 2 Methods

We developed this systematic map by following guidelines from the Collaboration for Environmental Evidence (CEE 2022) and the ROSES reporting framework (Haddaway et al. 2018). Our team included two faculty members and two graduate students, each experienced in oxygen mass transfer. Together, we defined the research questions, search strategy, and eligibility criteria. We also consulted additional academic experts to help identify key grey literature and benchmark studies.

### 2.1 Research Question and PECO Framework

The main research question for this systematic map is: What evidence is available about how chemical and biological surfactants affect oxygen mass transfer at the air–water interface? We used the PECO (population, exposure, comparator, outcome) framework to organize the question. Population refers to air–water interfaces in aquatic systems. Exposure includes chemical surfactants, such as SDS or linear alkylbenzene sulfonate (typical laboratory surfactants used to reduce surface tension), as well as biosurfactants, such as rhamnolipids (RHAs) or surfactants (surface‐active compounds produced by organisms), in the liquid phase. Comparators are clean water or baseline conditions without surfactants. Outcome covers oxygen mass transfer parameters, including the liquid‐side mass transfer coefficient (k_L_, a measure of how easily oxygen moves across the water boundary), volumetric mass transfer coefficient (k_L_a, the product of k_L_ and interfacial area a), and alpha factors (α, ratios comparing mass transfer with and without surfactants).

### 2.2 Search Strategy and Information Sources

We conducted comprehensive searches across two major bibliographic databases: Web of Science (Clarivate) and Scopus (Elsevier). Web of Science includes the Core Collection (from 1945), Preprint Citation Index, and SciELO Citation Index. Scopus covers content from 1960 onward. We developed the final search string step by step using the litsearchr package in R (Grames et al. 2019). This tool helped us identify keywords through text mining and co‐occurrence analysis of 32 benchmark articles. We further refined keywords using VOSviewer to analyze clustering (van Eck and Waltman 2010). The final Boolean search string included terms for population (air–water interface, gas–liquid interface, liquid phase, phase interface), exposure (surfactant concentration, surface active agent, anionic surfactant, biosurfactant, microbial surfactant), and outcome (oxygen transfer, k_L_, k_L_a, bubble column, gas absorption, Reynolds number). We used exclusion terms to filter out topics such as pulmonary, lung, wastewater treatment plant, or CO_2_. Searches took place in October 2023 (test list) and October 2024 (final list). We did not apply language restrictions during the search. However, we only screened full texts in English and Portuguese. We also searched organizational websites. These included Conservation International, International Water Management Institute, Plymouth Marine Laboratory, The Nature Conservancy, United Nations Environment Programme, and World Health Organization. We confirmed our search was comprehensive by retrieving all 32 benchmark articles from the test list.

### 2.3 Screening and Eligibility Criteria

We imported all records into Zotero to remove duplicates. Next, we transferred them to CADIMA software (Kohl et al. 2018) for systematic screening. Two reviewers (L.O. and D.R.) independently screened the titles and abstracts for the test list (*n* = 239). They reached a high level of agreement (Cohen's Kappa = 0.93, or 93% agreement). We considered studies eligible if they were empirical (laboratory experiments, field studies, or modeling), reported quantitative data on oxygen transfer, included clear exposure to surfactants at air–water interfaces, and measured at least one outcome variable. These outcome variables included k_L_, k_L_a, α, surface tension, or dissolved oxygen concentration. We excluded review articles, meta‐analyses, conference abstracts without full data, studies focused solely on wastewater treatment, liquid–liquid or liquid–solid interfaces, and studies focused solely on heat transfer.

Full‐text screening was independent for the test list (72 assessed, 49 retained) and by L.O. for the final list. Studies from 1963 to 2024 were included. Reasons for exclusion are in Data S5 and S6.

### 2.4 Data Extraction and Coding

LO extracted metadata using standardized forms. We collected bibliographic information (authors, year, journal, DOI), study design (laboratory or field setting, experimental apparatus, scale), and population characteristics (water type such as tap, distilled, seawater, or freshwater). We also collected exposure details, including surfactant identity (anionic, molecules with a negative charge; cationic, molecules with a positive charge; non‐ionic, molecules without a charge; amphoteric, containing both positive and negative charges; or biosurfactant, surfactants produced by living organisms) and concentration ranges. Outcome variables included k_L_, k_L_a, α, σ (surface tension), and a. We recorded explanatory or moderating variables, including hydrodynamic conditions (water movement and flow characteristics), temperature, salinity, pH, gas flow rate, and Reynolds number (a dimensionless number that expresses the flow regime). We grouped studies by surfactant type based on chemical structure. These included anionic (such as SDS, sodium lauryl sulfate [SLS], linear alkylbenzene sulfonate), cationic (such as cetyltrimethylammonium bromide [CTAB]), non‐ionic (such as Triton X‐100, Tween 80), amphoteric, and biosurfactants (RHAs, surfactant, lipopeptides). This approach addressed concerns about organizing by name instead of by properties.

### 2.5 Synthesis and Visualization

We identified knowledge clusters and gaps using quantitative bibliometric analysis and expert assessment. We used R software to create heat maps and cluster diagrams. These diagrams show relationships between water types and surfactant types. They also show outcome variables and experimental conditions, as well as patterns among independent and dependent variables. We also mapped co‐authorship networks to find geographic and institutional research clusters. We analyzed publication trends from 1963 to 2024 to see how research priorities have changed. All synthesis was done narratively, following systematic mapping standards. We did not use meta‐analysis or effect direction counts (James et al. 2016).

## 3 Results

The systematic search identified 403 records; after removing duplicates, 328 unique studies remained (81.39% retention). Reviewers showed agreement on which studies to include (Cohen's Kappa = 0.93). In total, 54 studies published from 1963 to 2024 were included. This process shows that the review was thorough and consistent, and the final dataset represents a wide range of published research from this period.

**FIGURE 1:**
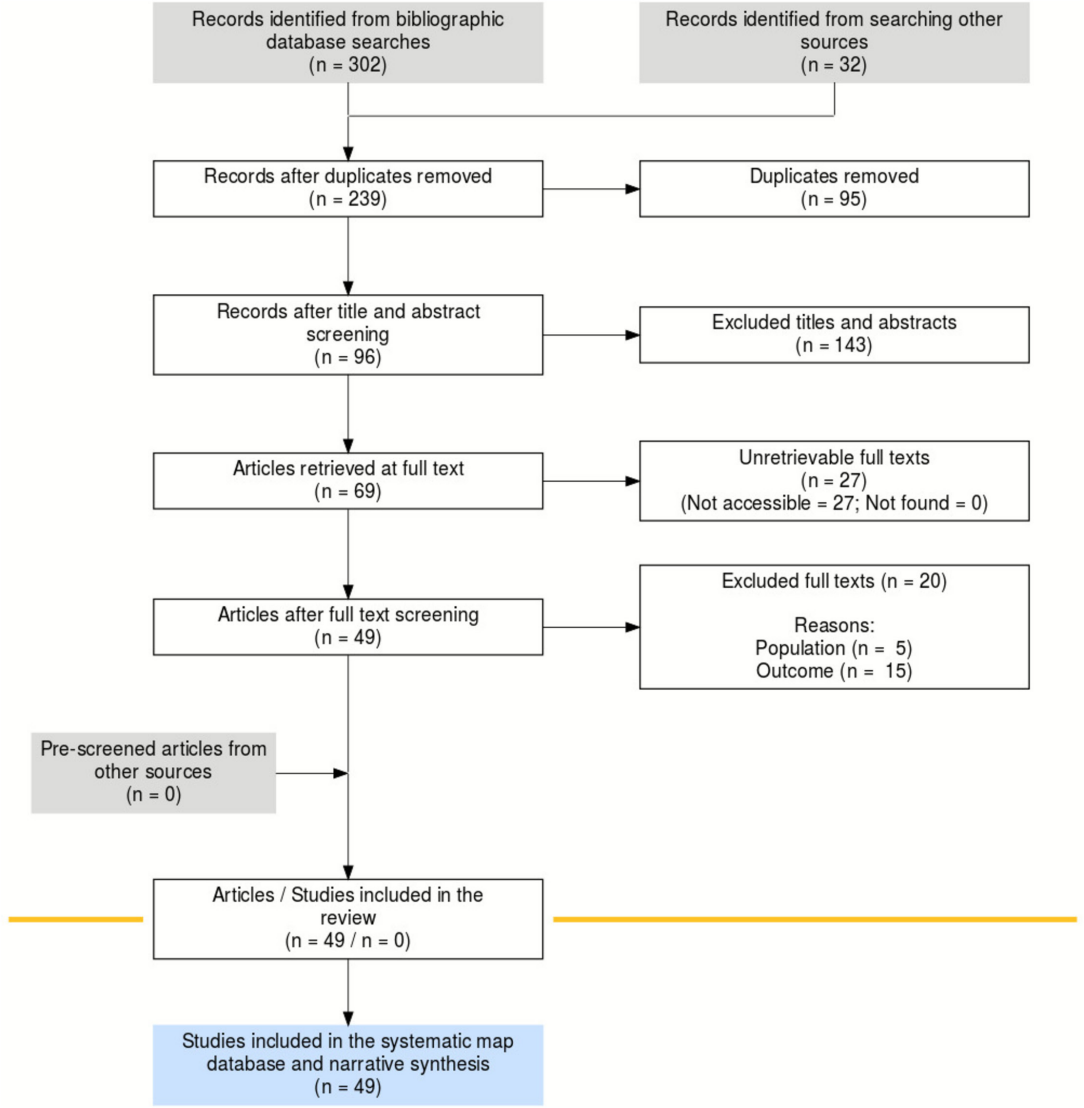
ROSES flowchart. Results of the search on digital indexing platforms using the search string defined based on the test list studies.

Figure 1 provides a visual summary of the search and screening process, illustrating how initial results were narrowed down to the final set of studies. This visual directly connects the search methods with the explanation of study inclusion, leading into the subsequent discussion of literature sources.

In addition to searching primary databases, grey literature was reviewed using Semantic Scholar. This search found 36 documents, of which 8 were eligible for screening. Because the Boolean operators limited the search, more studies were found on websites such as the Environmental Protection Agency and the Plymouth Marine Laboratory, which added three relevant articles. Experts also helped identify important methodological references, and all 32 benchmark articles were included. Together, these steps ensured the search was thorough and provided a base for evaluating the included populations and exposure types, which are discussed next.

Among the 54 included studies, most (74.07%) focus on gas–liquid interfaces, with the vast majority conducted in laboratory settings—only one study used field measurements. Another key gap is the lack of details in methodology reporting: although water was the most common sample (13 studies, 24.07%), information on factors such as pH, conductivity, and hardness was often missing. This lack of methodological details makes it difficult to reproduce results and weakens the study's conclusions. Identifying these gaps provides essential context on study populations and methodological strengths and weaknesses, setting the stage for the discussion of the investigated exposure types.

Of the 54 studies, 36 (66.67%) examined surfactants, and 14 of these also examined other types of contaminants. Seventeen studies (31.48%) focused only on non‐surfactant pollutants. Only three studies (5.56%) examined biosurfactants, which shows a clear gap in current research, even though biosurfactants are important for the environment. This gap highlights the need for more research on biosurfactant effects. The following breakdown shows how chemical and biosurfactant studies were distributed and introduces the outcome variables used to analyze oxygen transfer.

Most research focused on chemical surfactants. Anionic surfactants were found in 30.78% of studies, with SDS and SLS being the most common because they are readily available and have lower surface tension. Sodium dodecylbenzene sulfonate (SDBS) was used less often. Non‐ionic surfactants included Triton X‐100, Tween 80, Pluronic F68, and some alcohol‐based compounds. Cationic surfactants like CTAB, hexadecyltrimethylammonium chloride (HTAC), and similar compounds were also studied. Biosurfactants such as RHAs, saponins (SAPs), and lipopolysaccharides (LPSs) were less toxic but cost more than synthetic options. Other contaminants studied included chelating agents (like EDTA and citric acid), alcohols (such as methanol, ethanol, and butanol isomers), humic substances, hydrocarbons, and antifoaming agents. Since these chemicals can interact and affect interfacial dynamics, and now that the types of contaminants and surfactants have been described, the next section discusses the outcome variables for oxygen transfer.

The studies measured oxygen transfer efficiency using k_L_ and k_L_a, which are related by the equation k_L_a = k_L_.a. The main finding is that surfactant contamination lowers both coefficients. This decrease affects how well the liquid film and the interfacial area transfer oxygen, showing how contaminants can reduce system performance.

Surfactants reduced both k_L_ and k_L_a by forming static layers at the interface, slowing the renewal of the liquid film. Ionic surfactants affected electrical conductivity and oxygen transport in ways that differed from non‐ionic surfactants. Only one study has examined the reaeration coefficient (k_2_), which matters for environmental modeling. More research is needed to relate these results to natural waters. Given these findings, it is essential to further examine how surfactants impact other measurements, such as the alpha factor (α) in contaminated water systems.

Alpha factors (α) indicate the ratio of the overall k_L_a in contaminated versus clean water. In wastewater systems, α values ranged from 0.3 to 0.9, signaling a significant reduction in oxygen transfer efficiency. As surfactant concentration increased, surface tension decreased, and oxygen diffusivity (D) changed similarly to k_L_ in contaminated systems. Together, these findings indicate that surfactants broadly impact mass transfer, reinforcing their environmental relevance as discussed above.

A total of 54 studies published from 1963 to 2024 were included for data extraction and synthesis (detailed study characteristics, key findings, and limitations for all 54 studies are provided in Table S1). We found that surfactants, which have both water‐attracting and water‐repelling properties, consistently slow the rate at which oxygen moves from air into water. The extent and reasons for this depend on the pollutant, the water type, and the experimental setup. The review highlights trends, open questions, and research methods that shape current understanding and future research.

Both k_L_ and k_L_a decrease as surfactant concentration increases. This shows that surfactants create barriers at the water's surface, reducing turbulence and slowing oxygen transfer. Depending on the type and amount of surfactant, k_L_ dropped by 20%–70%. SDS and SLS were most often studied, likely because they are easy to obtain and produced k_L_values ranging from 1.60 × 10^−4^ to 3.52 × 10^−3^ m/s. The structure of the surfactant mattered: longer‐chain surfactants like sodium tetradecyl sulfate (STS) caused larger drops in k_L_ at lower concentrations than shorter‐chain SDS, suggesting they stick to the surface more. Triton X‐100 and Tween 80 were used in 13% of studies, while CTAB and HTAC were used in 7.4%, indicating that research focused on a small group of chemicals. Only three studies examined biosurfactants, natural compounds produced by microbes. These caused smaller drops in k_L_ and k_L_a than synthetic surfactants at the same concentrations, so they interfered less with oxygen transfer while still functioning as surfactants. But biosurfactants were tested only in simple water, so we do not know how they function in more complex environments. Because they break down more readily and are less toxic, biosurfactants could be better suited to sustainable industrial applications that require effective oxygen transfer, so more research is needed. Hierarchical clustering analysis revealed that most studies emphasized surfactant concentration (53 studies) and hydrodynamic conditions (41 studies). Other critical parameters received little attention. For example, the Reynolds number was considered in only four studies, despite its importance for predicting scale‐up and laminar‐to‐turbulent transitions. Similarly, fewer than five studies examined pH, viscosity, and atmospheric pressure. This gap hinders the evaluation of effects on surfactant ionization, micelle stability, and diffusion. In addition, 21 studies assessed bubble‐dynamics variables, such as diameter, rise velocity, and coalescence, in isolation. This isolated approach fragmented the understanding of interfacial renewal mechanisms.

Most of the 54 studies were conducted in labs and examined 18 types of water. The main types were tap water, generic water, distilled water, and deionized water. Only one study used seawater, and none studied nutrient‐rich lakes or streams affected by wastewater. About a quarter of the studies using generic water did not report essential details such as pH, conductivity, hardness, or dissolved organic carbon, making it hard to reproduce their results. Heat map analysis showed that nearly all water‐surfactant combinations have not been studied. Field studies were rare, with Anikiev et al. (1988) being a key example. Even though early work was done by Aiba and Toda (1963), Hwang and Stenstrom (1985), and van der Meer et al. (1992), there is still no standard way to do these experiments. Different measurement methods make it hard to compare results across studies. Research has picked up since 2000, with most studies published after that year. Still, researchers do not collaborate much, as shown by weak co‐authorship networks.

In this map, author‐reported labels such as “tap water,” “generic water,” “clean water,” and “unpolluted reference water” were treated as operationally equivalent clean‐water matrices whenever they were used as standard reference conditions in aeration tests. Conversely, labels that implied chemically distinct environments (e.g., seawater, filtered groundwater, or sodium chloride solutions) were retained as separate water types. This approach avoids artificially inflating the number of water types due to synonymous naming while preserving differences relevant to oxygen transfer.

There are common limitations in these studies. Few used designs that examined how pH, temperature, surfactant type, and Reynolds number interact. The concentration ranges tested were often too narrow to see effects below and above the critical micelle concentration. Bubble contamination as bubbles rise was rarely studied. Necessary modeling tools, such as the Sherwood, Schmidt, and Rayleigh numbers, were seldom used. Publication bias also affected the literature. These gaps make it hard to apply lab results to real‐world systems, such as wastewater treatment or natural water bodies. More field testing and standard methods are needed.

Hierarchical clustering showed that surfactant concentration and hydrodynamics were the main explanatory variables, each appearing in 53 and 41 studies, respectively. The analysis also identified four main clusters of variables.

### 3.1 Explanatory Variables Influencing Oxygen Transfer

**FIGURE 2:**
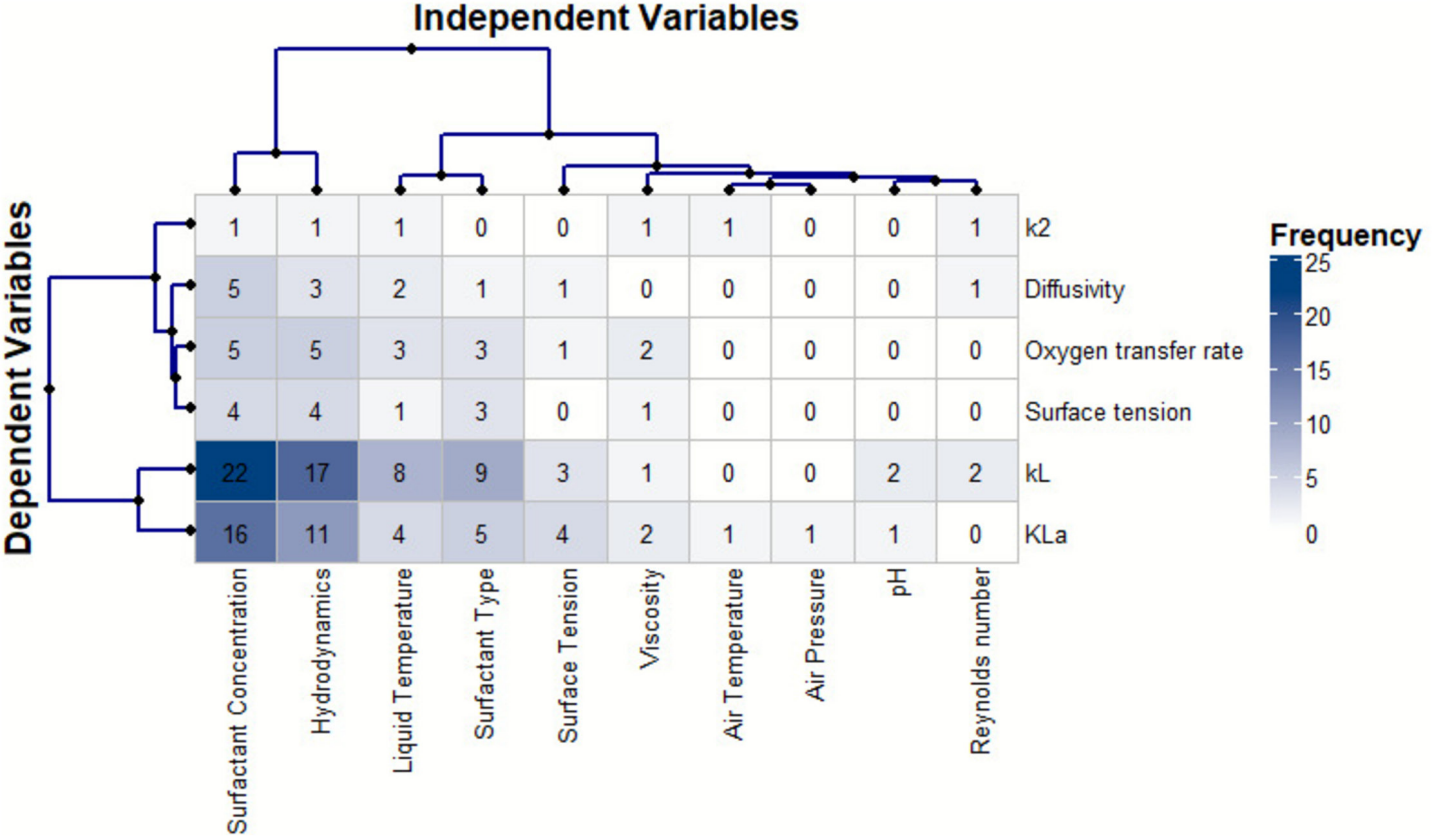
Heat map and hierarchical cluster dendrograms showing relationships between independent variables (surfactant concentration, hydrodynamics, temperature, surfactant type, etc.) and dependent variables (k_L_, k_L_a, oxygen transfer rate, surface tension, diffusivity, k_2_). Clusters reveal dominant variable associations and research gaps.

In Figure 2, the variable clusters identified in this analysis warrant further attention. Cluster 1, which includes k_L_ and k_L_a, had the most variables, showing their importance in measuring oxygen transfer efficiency. Both coefficients showed similar patterns and were closely linked to surfactant concentration (38 studies) and hydrodynamic conditions (28 studies), which are the main factors affecting oxygen transfer in water. Surfactant type (14 studies) and liquid temperature (12 studies) showed moderate associations, while surface tension was reported in 7 studies. Variables such as viscosity, pH, air temperature, air pressure, and Reynolds number were rarely studied, indicating clear gaps in the research. In Cluster 2, oxygen transfer rate and surface tension were each linked to surfactant concentration (9 studies), hydrodynamics (9), surfactant type (6), and liquid temperature (4). Cluster 3 focused on diffusivity, with most studies looking at surfactant concentration (5), hydrodynamics (3), and liquid temperature (2). This cluster stands out for its focus on the fundamental physical processes underlying oxygen transport in fluids. Viscosity effects on oxygen transfer were reported in 7 diffusivity studies, more often than for other variables. Cluster 4, which covers k_2_, was studied only once (Ferreira et al. 2020), focusing on surfactant concentration, hydrodynamics, liquid temperature, air temperature, and viscosity, but not surfactant type, surface tension, pH, air pressure, or Reynolds number. This narrow focus shows a research gap and suggests that studies on reaeration tend to be very specialized.

Expanding on the influence of hydrodynamics, researchers used dimensionless numbers to describe hydrodynamic flow. The Reynolds number (Re), an important parameter for understanding turbulence, was included in only four studies. This limits how well the findings apply to systems with different flow properties. Lee and Saylor (2010) linked the Sherwood number (Sh) to Re and the Rayleigh number (Ra) to predict k_L_ on contaminated surfaces; their study does not address gas‐side oxygen depletion or microbial oxygen consumption. Similarly, Lebrun et al. (2022) compared the effects of the Schmidt number in pure and surfactant‐contaminated water.

In addition to hydrodynamic measurements, bubble dynamics variables, including population, size, diameter, rise velocity, drag coefficient, coalescence, and shape, were examined in 21 studies. For instance, Jimenez et al. (2014) established relationships among the bubble contamination angle, rise velocity, and oxygen concentration fields, demonstrating their influence on the transfer flux (F) and flux density (J).

**FIGURE 3:**
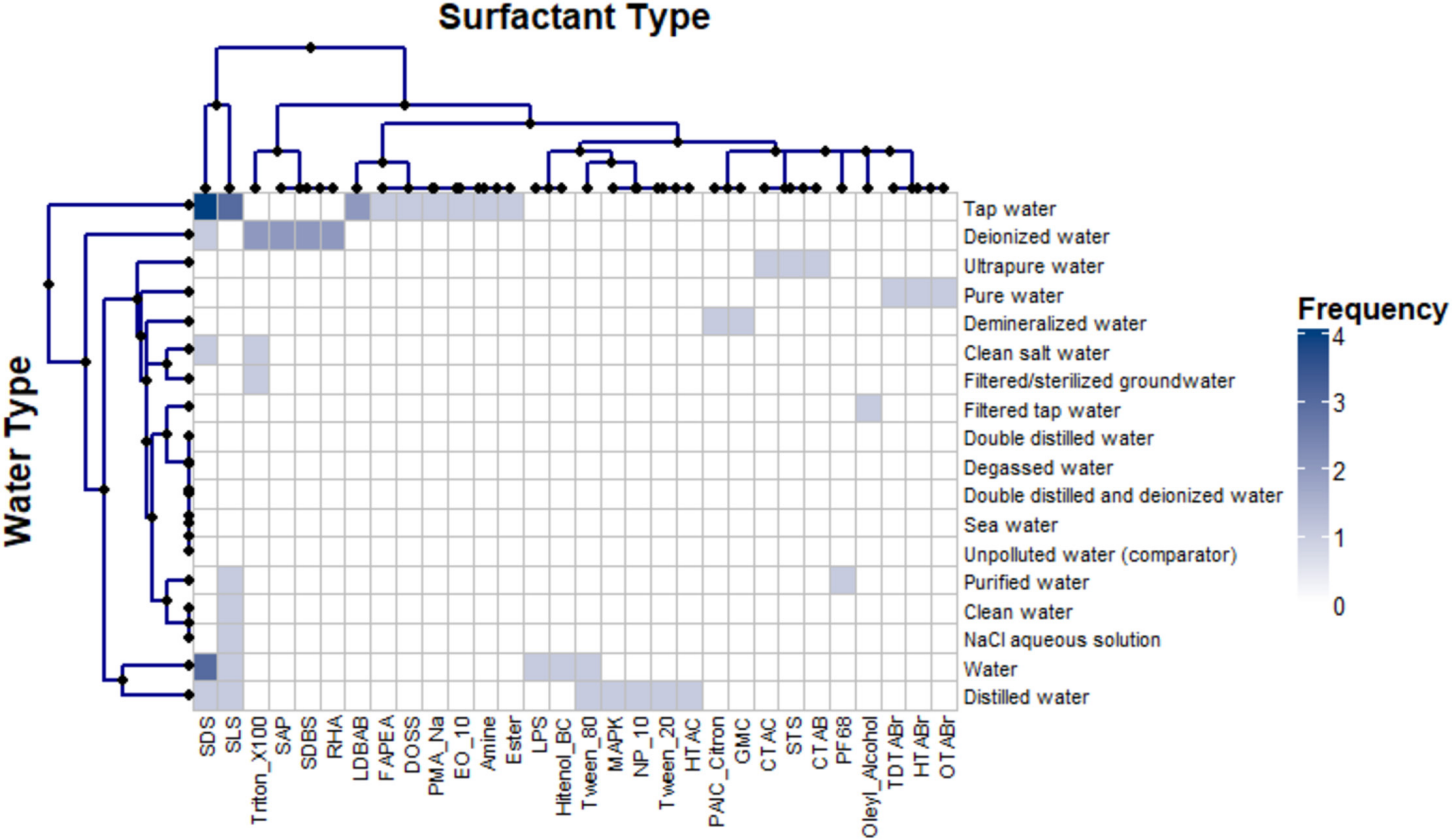
Heat map with row and column dendrograms illustrating the frequency of studies combining 18 water types with 30 surfactant types. Color intensity represents study frequency (0–4 occurrences). Dendrograms reveal clustering patterns and unstudied combinations.

Using the heat map patterns, a hierarchical analysis of the 18‐by‐30 data matrix for water and surfactant types (Figure 3) identified five clear water‐type clusters. Finding these clusters matters because it shows where research is concentrated and which water types are often studied, helping to set future research priorities. This analysis continues the clustering methods used earlier, maintaining consistency throughout the study.

Cluster 1 (tap, generic, distilled water) exhibited the surfactant diversity, including SDS (8), SLS (5), LDBAB (2), Tween 80 (2), and 13 additional surfactants each tested once. This cluster reflects the predominant research emphasis on accessible and standardized water types. Cluster 1, therefore, groups several author labels that effectively correspond to clean or tap water as used in standard aeration tests, rather than distinct environmental matrices.

Cluster 2 (deionized water) included both chemical surfactants, such as Triton X‐100 and SDBS, and biological surfactants, including SAP and RHA, each tested in two studies. This cluster represents controlled, high‐purity experimental conditions suitable for mechanistic investigations. Cluster 3 (ultrapure, pure, demineralised, purified, clean saltwater) utilized specialized surfactants with limited applications, such as PAIC Citron, GMC, cetyltrimethylammonium chloride (CTAC), STS, CTAB, TDTABr, HTABr, OTABr, and PF68, each examined in a single study. This cluster represents niche industrial or nanotechnology applications. No directly associated surfactants were identified for some water types, indicating research focused on non‐surfactant contaminants or baseline measurements. Cluster 5 (filtered tap, clean, sodium chloride solution, filtered groundwater) included water types tested with only one or two surfactants, such as oleyl alcohol, SLS, and Triton X‐100. This shows these areas are not well studied. Six groups mainly used SDS and SLS, both anionic surfactants, across six water types each. Biosurfactants such as RHA, SAP, and LPS were tested only in deionized or generic water, accounting for just 5.56% of studies. The heat map shows that 92.22% of possible water‐surfactant combinations have not been studied.

Even though many water bodies are essential for the environment and industry, surfactant studies on seawater, unpolluted water, double‐distilled water, filtered tap water, clean water, filtered groundwater, and sodium chloride solution are few, which limits the applicability. Biosurfactant studies are also limited, even though they are essential for the environment. RHAs and SAP have shown promising results, but insufficient testing across different water types prevents firm conclusions about their performance in real‐world conditions. Overall, studies rarely include variables like pH, Reynolds number, air humidity, atmospheric pressure, and viscosity. Including these will make models more accurate and valuable in the real world. The frequent use of synonymous labels for clean‐water conditions (“tap,” “clean,” “generic,” “unpolluted”) also introduces terminological variability that can hinder direct comparison between studies.

Kouzbour et al. (2023) identified the need to investigate the effects of bubble diameter at high gas flow rates. Lebrun et al. (2022) called for an improved understanding of surfactant adsorption/desorption kinetics to predict bubble contamination status. Ertekin et al. (2021) highlighted gaps in the effects of bubble‐size distribution on volume‐fraction corrections, lift forces in turbulent flows, and contaminant‐mediated bubble interactions. Weiner et al. (2019) noted insufficient understanding of bubble wake effects on oxygen concentration fields.

Most studies examined simple relationships and rarely combined factors such as pH, flow, viscosity, and surfactant type. This makes it harder to predict how complex systems behave and to fully understand oxygen transfer.

### 3.2 Co‐Authorship Networks and Research Collaboration

The co‐authorship networks from the 54 mapped studies (see Figures S1 and S2) reveal that most experimental evidence comes from a few main collaboration clusters. Some groups are more connected and help link different subfields. However, these patterns reflect only this specific set of studies and should not be interpreted as a ranking of researchers in the broader field.

The network's fragmented structure suggests that new methods and experimental protocols tend to remain within specific communities, with little exchange across fields such as marine biogeochemistry, environmental engineering, and interfacial physics. Because of this, similar problems, such as how surfactant films affect bubble dynamics and turbulence, are often studied separately in each field. Encouraging more collaboration between these areas could help develop shared methods and models that apply more broadly. Table S2 clusters show co‐authorship and methodological focus.

The Plymouth Marine Laboratory's 8th International Symposium on Gas Transfer identified additional scholars investigating gas transfer at air–water interfaces, focusing on physical processes, turbulence, and the effects of natural and synthetic surfactants (Jessup and Asher 2016). This group contributes significantly to biogeochemical gas exchange understanding but remains underrepresented in the systematic map corpus.

## 4 Discussion

This work is a systematic evidence map, not a narrative literature review. It uses a different method to catalog research, measure knowledge gaps, and identify themes without combining effect sizes (James et al. 2016; Collaboration for Environmental Evidence 2022).

Our systematic mapping approach stands out from traditional reviews in four main ways. First, it ensures reproducible evidence synthesis by using pre‐registered protocols (see Data S1), search strings that found all benchmark articles (32 out of 32), and dual‐reviewer screening with 93% agreement (Cohen's Kappa = 0.93). Narrative reviews, such as those by Jamnongwong et al. (2010), do not use these standards. Second, our quantitative gap analysis reveals that 92.22% of water‐surfactant combinations have not been tested, 94.44% of studies did not include biosurfactants, even though they are environmentally important, and 94.44% lacked field validation. Earlier reviews only mentioned these issues in general terms, without measuring them.

Third, we use new analytical tools, including hierarchical clustering of 18‐by‐30 water‐surfactant matrices to highlight research gaps (Figure 3), co‐authorship network analysis to show the divide between marine biogeochemistry and chemical engineering, and heat maps to show variable associations (Figure 2). Earlier syntheses did not use these visualization tools. Fourth, we offer practical guidance for stakeholders by turning abstract findings into clear research priorities. For example, we suggest biosurfactant dose–response curves in natural waters to support green chemistry policies, α‐factor measurements for biosurfactants to help optimize aeration costs (with potential energy savings of 10%–70% if biosurfactants reach *α* > 0.6, compared to 0.3–0.4 for synthetic surfactants as shown by Hwang and Stenstrom 1985), and field k_2_‐Reynolds number relationships for modeling dissolved oxygen in surfactant‐polluted streams (currently based on just one study: Ferreira et al. 2020).

These methodological and empirical contributions lay the groundwork for targeted meta‐analyses. These experimental designs address interaction effects such as pH, Reynolds number, and surfactant type, and field studies in less‐studied environments, including seawater, eutrophic lakes, and streams affected by agricultural runoff. The systematic map is a new contribution because it organizes 60 years of scattered research into a structured, searchable evidence base with clear research priorities. Narrative reviews cannot provide this foundation.

Only 5.56% of studies (3/54) involved field measurements, with 94.44% confined to controlled laboratory experiments using simplified water matrices (tap, distilled, deionized). While existing reviews acknowledge laboratory dominance, we demonstrate its consequences: the synergistic effects of humic substances with surfactants remain unexamined, and the effects of seawater ionic strength on biosurfactant CMC remain unexplored. The single k_2_ (reaeration coefficient) study limits its applicability to natural water‐body modeling. By systematically characterizing these limitations, 5.56% of studies (3 out of 54) included field measurements, while most research (94.44%) was conducted in labs using simple water types such as tap or distilled water. Other reviews have noted this focus on lab work. Still, we show that questions—such as how humic substances interact with surfactants or how seawater affects biosurfactant properties—have not been studied. Only one study has looked at the reaeration coefficient, which limits how well we can model real water bodies. By clearly outlining these gaps, we help guide future research, especially on how biosurfactants work in complex environments, how to measure their effects in pilot‐scale systems, and how different factors, such as pH and water flow, interact with surfactant chemistry. These steps are needed to turn decades of research into practical tools for managing water quality and responding to climate challenges. However, more relevant compounds for real‐world pollution are being overlooked. To move the field forward, researchers need to test how biosurfactants affect oxygen transfer in different types of water. Working together on targeted studies will help address both economic and technical challenges and enable the broader use of biosurfactants.

### 4.1 The Biosurfactant Deficit

The systematic map showed a clear research gap: only 5.56% of studies (3 out of 54) focused on biosurfactants, despite their well‐known environmental benefits over synthetic surfactants. Biosurfactants, such as RHAs, SAPs (plant‐derived glycosides), and LPSs (from bacterial cell walls), are more biodegradable and less toxic than typical anionic surfactants. Anionic surfactants include SDS and SLS. SDS and SLS were reported in nearly a third of studies. This is likely due to their availability and long‐term research use. However, this focus has left out compounds that are now gaining importance in real‐world pollution cases.

There are several reasons why biosurfactants have not been studied as much. Producing them on a large scale is expensive. Pure biosurfactant standards are costly in experiments. Their structures vary widely, including glycolipids, lipopeptides, and polymers. These differences make it harder to develop standard testing methods. (Glycolipids have a carbohydrate and a lipid; lipopeptides have a lipid and a peptide; polymers are large molecules built from repeating units.) This complexity may discourage researchers who are more familiar with well‐defined chemical surfactants. Some evidence shows that biosurfactants reduce k_L_ and k_L_a less than synthetic anionic surfactants at the same concentrations. This may result from differences in how they adsorb and pack at surfaces. This points to a trade‐off: biosurfactants might disrupt oxygen transfer less while still acting as surfactants. However, this idea needs more testing in different types of water.

The alpha factor (α) is the ratio of k_L_a in contaminated water to k_L_a in clean water. Here, k_L_a means the volumetric oxygen transfer coefficient, which is essential in wastewater treatment engineering. However, only some reviewed studies measured α directly. Early research by Eckenfelder (1970) and later by Stenstrom and others showed that α is essential for adjusting oxygen transfer efficiency in activated sludge systems and typically varies from 0.3 to 0.9. Surfactants reduce surface tension and appear in household and industrial products. They can minimize aeration performance. The exact value depends on surfactant concentration, temperature, and mixing. These values directly impact energy costs in large wastewater treatment plants.

To fully leverage laboratory findings for real‐world impact, future research should systematically measure α across biosurfactant types, concentrations, and relevant environmental conditions, including natural waters such as rivers and lakes. Doing so will not only enhance the practical design of aeration systems using green surfactants but also improve predictions of dissolved oxygen recovery after biosurfactant pollution in the environment. Researchers are encouraged to incorporate α as a standard parameter in both lab and field studies to bridge the gap between fundamental science and applied engineering.

### 4.2 Laboratory Dominance and Water Matrix Simplification

Most studies in this field rely on laboratory experiments. Only one field study examines seawater oxygen transfer under petroleum contamination. Lab settings help isolate variables and clarify mechanisms, but they miss natural complexities. These include changing turbulence, suspended particles, microbial films, sunlight‐driven breakdown, and interactions between pollutants. Using tap, distilled, or deionized water in experiments also limits how well the results apply to real environments, such as nutrient‐rich lakes, estuaries, or streams affected by wastewater.

This focus on lab studies limits the extent to which the results apply to real‐world situations. Humic substances are common in natural waters and can reduce oxygen transfer rates by up to 17%, but their combined effects with surfactants have not been studied. Seawater's salt content also alters surfactant behavior, potentially affecting results compared to freshwater. Most water‐surfactant combinations have not been tested. This leaves a significant gap in our knowledge. Future research should use mesocosm and field studies, employing tools such as oxygen microsensors and environmental DNA. These efforts can help determine whether lab findings hold in real‐world environments.

Analysis shows most studies focus on how much surfactant is present and how water moves. Other factors get little attention. Only three studies examined pH, even though it affects how surfactants interact with surfaces. The Reynolds number helps us understand water flow and how to apply lab results in real‐world situations, but it was reported in only four studies. This matters because it affects how oxygen moves in water, whether in nature or factories.

In most laboratory experiments, water temperature is controlled and k_L_ or k_L_a values are reported or normalized to a reference temperature (typically 20°C), because temperature strongly affects oxygen solubility. This standardization partly masks temperature effects in the reported coefficients, even though water temperature remains physically important for mass transfer. Similarly, total pressure is usually close to atmospheric pressure. It is measured to calculate the saturation concentration of dissolved oxygen. Still, it is rarely varied as an independent design variable in the experiments mapped here, which limits our ability to quantify its influence from the published data.

The near‐absence of atmospheric pressure and air humidity as explicit explanatory variables limits the model's predictive accuracy, especially in high‐altitude or variable‐climate scenarios. Ferreira et al. (2020) showed that air temperature and viscosity influence the reaeration coefficient (k_2_). However, subsequent models have not integrated this finding. Viscosity effects were examined in seven studies focused on diffusivity, but they were seldom analyzed with k_L_ or k_L_a. This is notable because viscosity directly affects boundary‐layer thickness and convective mixing.

Studies on bubble behavior are often disconnected, with factors such as size, speed, and merging typically examined separately rather than together. Lebrun et al. (2022) noted that we need a better understanding of how surfactants stick to and detach from bubbles to predict contamination as bubbles rise, which calls for combined fluid dynamics and thermodynamics models. Ertekin et al. (2021) noted gaps in how we measure lift force in turbulent water and how bubbles interact when surfactants are present. Solving these problems will require advanced techniques such as high‐speed imaging, laser‐based oxygen mapping, and computer models validated against microfluidic experiments.

Most research on oxygen transfer has focused on SDS and SLS, which were widely used in industry decades ago. Today, non‐ionic surfactants such as alcohols and alkylphenol ethoxylates are more common. However, they appear in only a small fraction of studies. Cationic surfactants are found in products such as fabric softeners and disinfectants, but are also rarely studied. This mismatch between what is learned and what is found in the environment makes it harder to assess risks and develop sound policies.

We lack enough data on biosurfactants. This makes it hard to support green chemistry policies that encourage biodegradable options. To set appropriate discharge limits, we need more information on how biosurfactants such as RHAs, surfactants, and sophorolipids affect oxygen transfer in rivers exposed to farm runoff or industrial waste. While biosurfactants appear to reduce oxygen transfer less than SDS does, this needs to be confirmed. Future studies should test different concentrations, water types, and turbulence levels that match real aquatic environments.

In wastewater treatment, insufficient data on the α‐factor for biosurfactants makes it hard to optimize aeration. This affects both industrial and household wastewater treatment. If biosurfactants have an α‐factor above 0.6, compared to about 0.4 for regular surfactants, large‐scale energy savings could be possible. However, some biosurfactants may form stubborn surface films at low concentrations, reducing their environmental benefits by causing operational problems. To address these issues, we need pilot‐scale studies using real wastewater rather than lab‐made water.

A look at the co‐authorship patterns (Figure S1) shows that most of the mapped studies come from a few concentrated clusters, with limited overlap between research communities. For instance, the group of studies on wastewater treatment and engineered aeration is disconnected mainly from work on gas transfer in the open ocean. The Plymouth Marine Laboratory symposium on gas transfer at water surfaces, for example, reveals an active marine community working on surfactant films. Still, this body of work is only partially captured in our 54‐study corpus. Marine ecologists, oceanographers, and environmental engineers often address similar interfacial processes, yet they tend to publish in separate venues and share little methodological detail across fields.

Bridging these communities could improve experimental design and data availability by encouraging shared protocols, co‐located measurements, and cross‐validation of models. For example, techniques used in coastal and open‐ocean studies (such as eddy‐covariance flux towers or robotic platforms equipped with oxygen sensors) could be adapted to quantify surfactant effects in rivers, reservoirs, and wastewater treatment systems, provided they are calibrated for different water types. Likewise, advances in process engineering, such as high‐resolution bubble imaging and controlled turbulence in reactors, could inform how oceanographers and limnologists parameterize gas transfer in the presence of surfactant films.

### 4.3 Limitations and Future Directions

There are some limitations to this systematic map. We decided not to use “surface tension” as a main search term to keep the number of results manageable. This choice may have led us to miss studies where oxygen transfer was not the primary focus. We also limited our search to English and Portuguese. As a result, we did not include research published in Chinese, Japanese, or German, which means some early work from Europe and Asia might be missing. In addition, only one reviewer screened 18.52% of studies at the full‐text stage, which could introduce selection bias. Still, the high Kappa agreement (0.93) at the title and abstract stage shows that our process was consistent.

Problems in the current evidence base further compound these limitations in our review. For example, about a quarter of studies use the term “water” without giving details, such as pH, conductivity, or dissolved organic carbon. Without this information, it is hard to reproduce results or perform a meta‐analysis. Furthermore, there is no standard method for measuring k_L_: some studies use oxygen probes, while others employ chemical methods or pressure decay. This variety makes it challenging to compare results. To address these issues, future reviews should encourage studies to report water characteristics, Reynolds numbers, and the purity or source of surfactants. None of the experimental studies included in this map explicitly quantified gas‐phase oxygen depletion or microbial oxygen consumption, so these mechanisms are discussed only as plausible hypotheses rather than documented effects.

To address these gaps, future research should focus on studying biosurfactant doses at realistic levels in natural waters and on measuring the α‐factor defined as the ratio of oxygen transfer efficiency in process water to clean water, for bio‐based surfactants in pilot‐scale reactors. In addition, research should jointly test pH, Reynolds number (a measure of flow turbulence), temperature, and surfactant type, and evaluate k_L_–Re relationships in real rivers and lakes with known surfactant pollution. Furthermore, combining computer modeling and experiments can help elucidate bubble‐surfactant interactions outside the lab. By prioritizing these areas, oxygen‐transfer research can advance toward the development of practical models to improve environmental management.

## 5 Conclusions

This study presents a systematic evidence map of 54 studies from 1963 to 2024, creating the first thorough, protocol‐based synthesis of how surfactants affect air–water interfaces and setting priorities for environmental management and wastewater treatment.

First, we found that chemical surfactants typically reduce oxygen transfer coefficients (k_L_, k_L_a) by 20%–70%. Anionic surfactants like SDS and SLS accounted for about 31% of the studies, not because they are common in the environment, but because they are readily available. Longer‐chain surfactants, such as STS, reduced k_L_ more sharply and at lower concentrations than shorter‐chain types. This shows that molecular structure affects how surfactants block oxygen transfer (Lebrun et al. 2022; Bai et al. 2024). In wastewater treatment, alpha factors (α) ranged from 0.3 to 0.9, which means aeration energy costs can rise by 10%–70% (Hwang and Stenstrom 1985; Rosso and Stenstrom 2006). Still, only some studies measured α directly, thereby missing an opportunity to connect research to engineering practice.

Second, biosurfactants are still rarely studied—only 3 of 54 studies examined these microbial compounds, even though they break down more easily and are less toxic than synthetic surfactants. Early results show that biosurfactants, such as RHAs and SAPs, reduce k_L_ less than SDS at the same concentrations (Bai et al. 2024; van der Meer et al. 1992). This points to their commitment to green chemistry, especially in oxygen‐transfer processes. But so far, tests have used only lab setups with deionized or generic water, so we cannot say how biosurfactants perform in real‐world conditions, such as seawater or wastewater. This lack of data makes it hard for regulators to set safe discharge limits for bio‐based industrial waste, since we do not have enough information on how biosurfactants behave across different pH, salinity, and turbulence levels.

Third, we found that most studies have limitations for real‐world use: over 96% were done in labs, and only one was a field study (Anikiev et al. 1988). Most research used tap water, generic water, or distilled water, while real environments—such as lakes, estuaries, and streams affected by wastewater—were rarely studied. Our analysis showed that almost all combinations of water and surfactant types have not been tested, leaving a knowledge gap. Factors like Reynolds number, pH, and air pressure were rarely included. Because of this, it is hard to build models that predict surfactant behavior under different real‐world conditions.

Fourth, we noticed that researchers in this field often work in separate groups, which slows the development of new methods. Marine biogeochemists focus on natural surfactant films, while chemical engineers study synthetic surfactants in industrial settings, but they rarely collaborate. We suggest that bringing these groups together could lead to better methods, such as using marine field tools in freshwater studies and applying engineering models to ocean research.

We suggest five main research priorities to help move oxygen transfer studies from just describing results to making predictions. First, create dose–response curves for biosurfactants at real‐world concentrations in natural waters, and measure alpha factors directly. Second, run pilot‐scale studies treating bio‐based industrial waste with domestic sewage to determine whether energy can be saved. Third, design experiments to test how pH, temperature, flow rate, and surfactant type interact. Fourth, do field tests in rivers and lakes with known surfactant pollution, using sensors and DNA tools. Fifth, combine computer modeling and lab experiments to study how bubbles and surfactants interact in ways that are hard to observe directly.

Closing these research gaps will take more funding and better methods. We recommend that all studies report key water details, Reynolds numbers, and surfactant sources to facilitate future comparisons. Our review shows that while we know a lot about how chemical surfactants block oxygen transfer, new issues, such as biosurfactants and green chemistry, require urgent attention. We believe our work gives a solid base for setting research priorities that support better water modeling, wastewater treatment, and sustainable industry.

## Author Contributions

**Luciano de Oliveira:** conceptualization, data curation, formal analysis, investigation, methodology, project administration, software, supervision, visualization, writing – original draft. **Diana Rosa dos Reis:** formal analysis, investigation, methodology, software, writing – original draft. **Sérgio Botelho de Oliveira:** conceptualization, methodology, supervision, validation, writing – review and editing. **Klebber Teodomiro Martins Formiga:** conceptualization, funding acquisition, methodology, resources, supervision, validation, writing – review and editing.

## Funding

This work was supported by the Coordenação de Aperfeiçoamento de Pessoal de Nível Superior, Financiadora de Estudos e Projetos, and Conselho Nacional de Desenvolvimento Científico e Tecnológico.

## Conflicts of Interest

The authors declare no conflicts of interest.

## Supporting information


**Data S1:** Supporting information.

**Data S2:** Supporting information.

**Data S3:** Supporting information.

**Data S4:** Supporting information.

**Data S5:** Supporting information.

**Data S6:** Supporting information.

**Data S7:** Supporting information.

**Data S8:** Supporting information.

**Data S9:** Supporting information.

**Figure S1:** Co‐authorship network visualization showing collaboration clusters among principal authors.

**Figure S2:** Temporal distribution of publications by research groups, highlighting evolving partnerships and productivity trends.

**Table S1:** Detailed study characteristics, key findings, and limitations.

**Table S2:** Clusters de coautoria e focos metodológicos.

## Data Availability

The data generated or analyzed during this study are included in this submitted article.
