# Supplementary material for: Systematic Map of Chemical and Biological Surfactant Effects on Oxygen Mass Transfer at the Air–Water Interface
Source: Water Environ Res. 2026 Jan 14;98(1):e70271. doi: 10.1002/wer.70271 (PMC12802822; doi:10.1002/wer.70271)
Supplement: Supplementary file 10 — Figure S1: Co‐authorship network visualization showing collaboration clusters among principal authors. [file WER-98-e70271-s011.pdf]

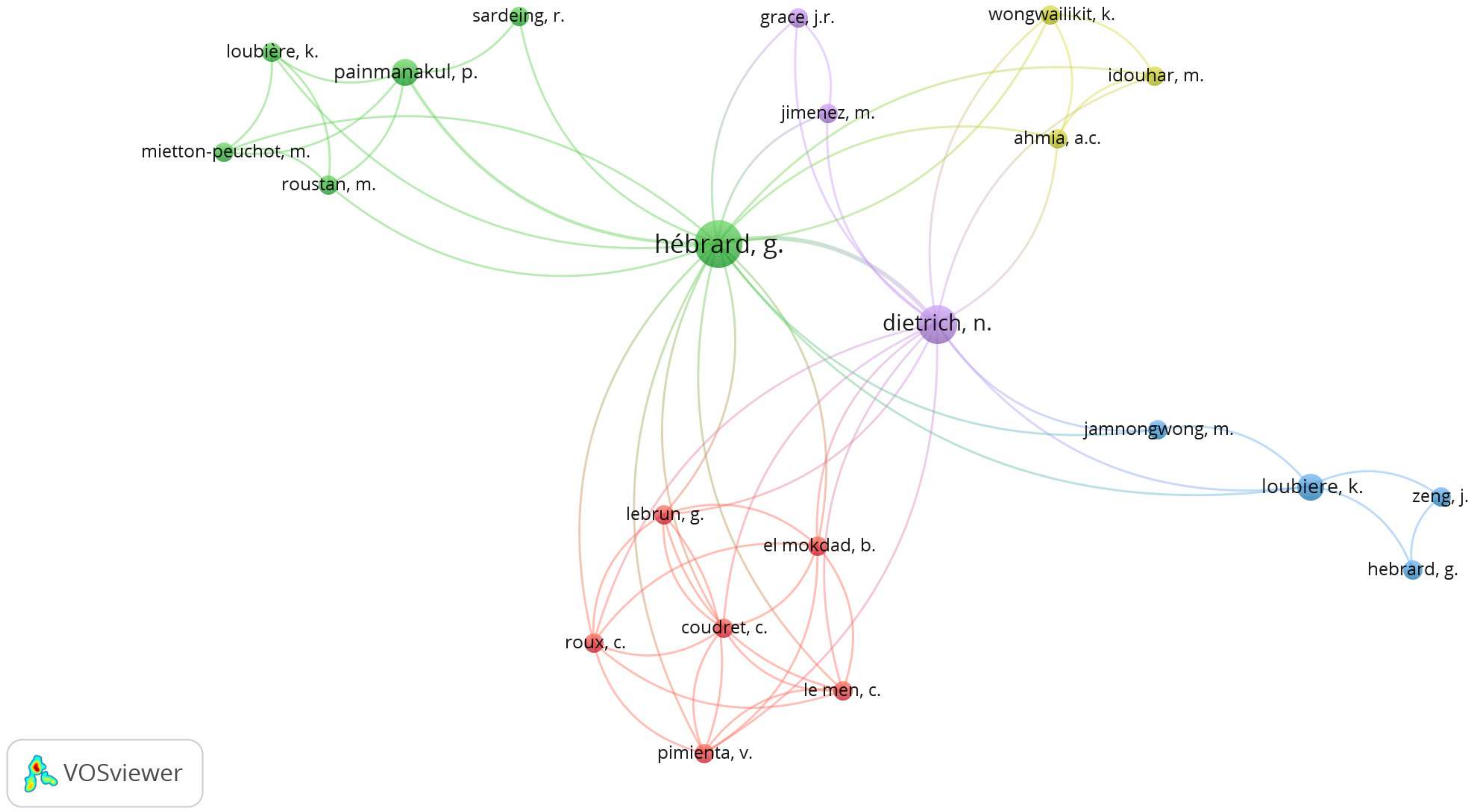

Figure S1. Co-authorship network visualisation showing collaboration clusters among principal authors
