# Supplementary material for: Systematic Map of Chemical and Biological Surfactant Effects on Oxygen Mass Transfer at the Air–Water Interface
Source: Water Environ Res. 2026 Jan 14;98(1):e70271. doi: 10.1002/wer.70271 (PMC12802822; doi:10.1002/wer.70271)
Supplement: Supplementary file 11 — Figure S2: Temporal distribution of publications by research groups, highlighting evolving partnerships and productivity trends. [file WER-98-e70271-s007.pdf]

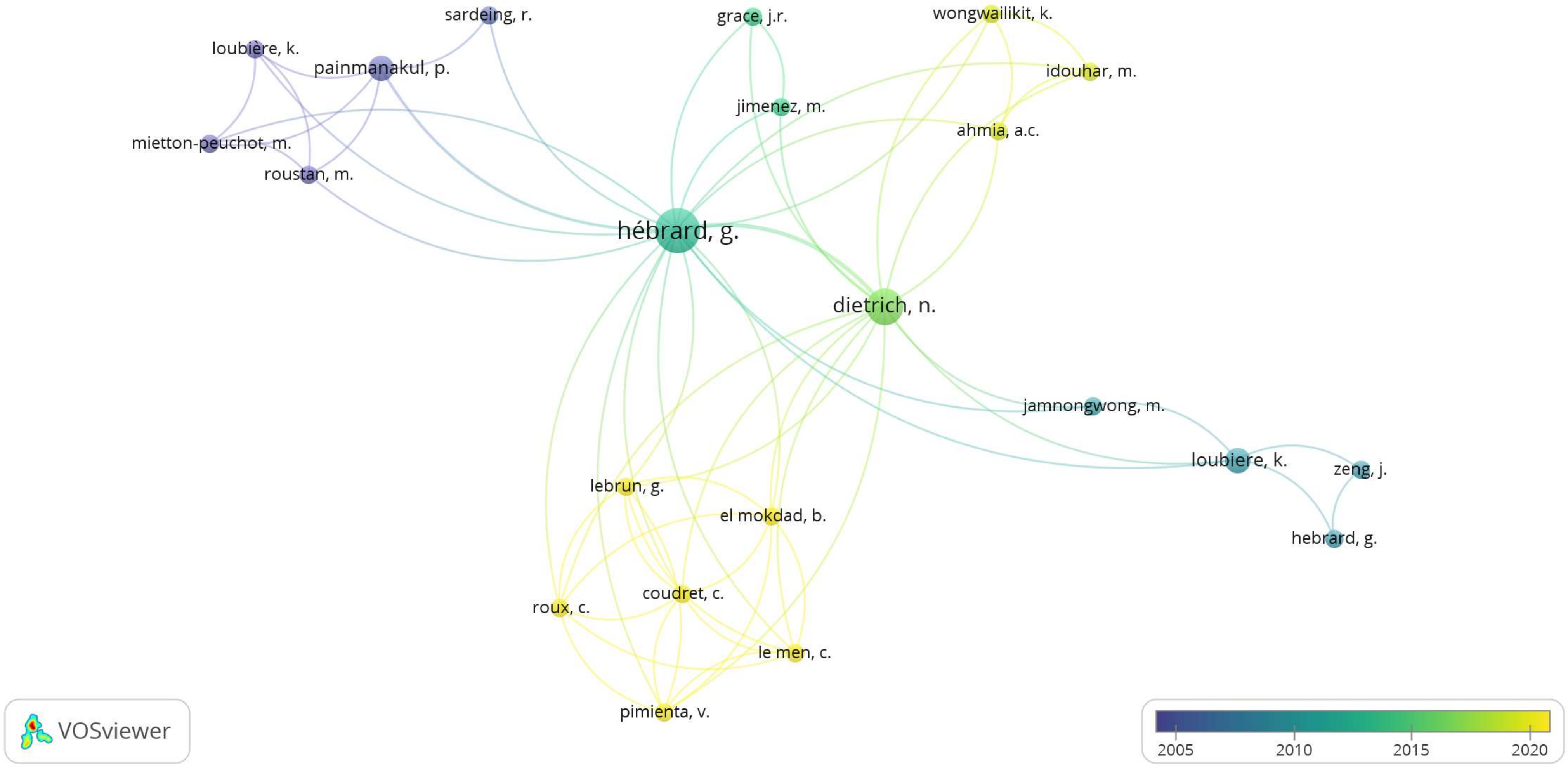

Figure S2. Temporal distribution of publications by research groups, highlighting evolving partnerships and productivity trends
